# Percutaneous treatment of acute axillary artery occlusion after percutaneous coronary intervention: A case report

**DOI:** 10.1002/ccr3.4338

**Published:** 2021-06-09

**Authors:** Akihiro Umeno, Shigeyasu Tsuda

**Affiliations:** ^1^ Division of Cardiology Kita‐Harima Medical Center Ono city Japan

**Keywords:** catheter‐induced arterial injury, endovascular treatment, JR guide catheter, self‐expandable stent

## Abstract

The JR guide catheter is preferred for operability; however, we should pay more attention to the guide catheter in the case of radial artery approach with severe vessel tortuosity especially in patients with hypertension or in older female patients.

## Graphical abstract

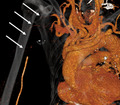

## 1 INTRODUCTION

Iatrogenic catheter‐induced arterial injury may cause ischemic upper extremity disease, but such cases are rare. There are two reports on complications during transcatheter aortic valve replacement^1^ and shoulder arthroplasty,^2^ both of which were treated by the surgical or interventional treatment. Transluminal angioplasty is another treatment option and shows excellent upper limb salvage for traumatic cases,^3^ but the reports on endovascular treatment (EVT) for iatrogenic cases are rare.

We present a case of right axillary artery occlusion, after percutaneous coronary intervention (PCI), treated with endovascular stent grafting.

## 2 CASE

An 88‐year‐old woman with a history of critical limb ischemia and a chief complaint of intermittent chest pain for 3 days was transported to the emergency room. ST elevation in leads III and aVF was displayed on an electrocardiogram, and laboratory data showed elevation of troponin I (442 pg/mL). Emergency coronary angiography (CAG) through her right radial artery access showed right coronary artery stenosis. We tried to change JR 4 Fr angiographic catheter to RU 6 Fr guide catheter for percutaneous coronary intervention. However, her axillary artery was so tortuous that catheter maneuver in it was so difficult.

We pushed against resistance several times but could not pass over it, and the catheter got kinked. Using JR 6 Fr did not work and got kinked, too. Therefore, we changed the right femoral artery access and successfully placed two drug‐eluting stents (3.5 × 15 mm and 4.0 × 15 mm) in her stenotic lesion of coronary artery.

**FIGURE 1:**
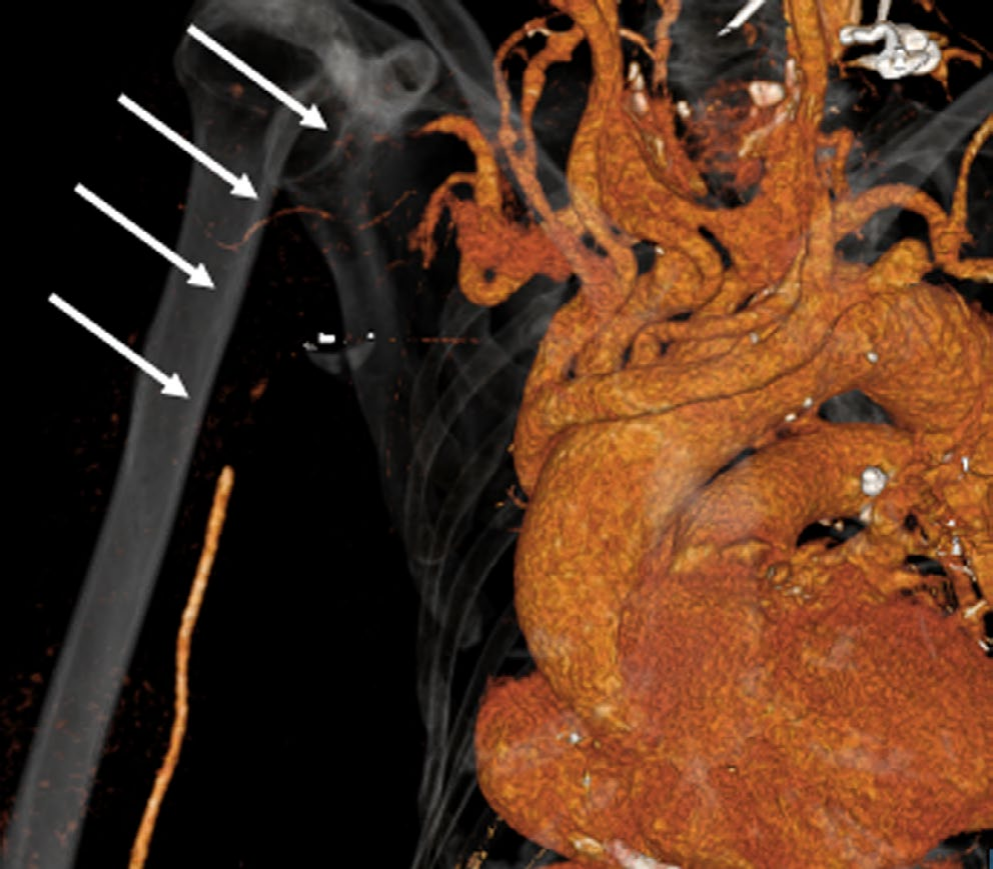
Three‐dimensional computed tomographic angiography revealed an occlusion lesion from the axillary artery to the brachial artery

Her chest pain reduced after the PCI, but she complained of slight pain in her right hand during the procedure. We prescribed acetaminophen, but her symptoms did not resolve. On the next day, the patient felt numbness and exhibited impaired skilled movement in the right arm. Physical examination showed nonpalpable right radial and brachial pulses, indicating advancing ischemia. We performed three‐dimensional computed tomographic angiography, which revealed an obstructive lesion from the axillary artery to the brachial artery. Therefore, we decided to perform urgent revascularization (Figure 1).

## 3 PROCEDURE

**FIGURE 2:**
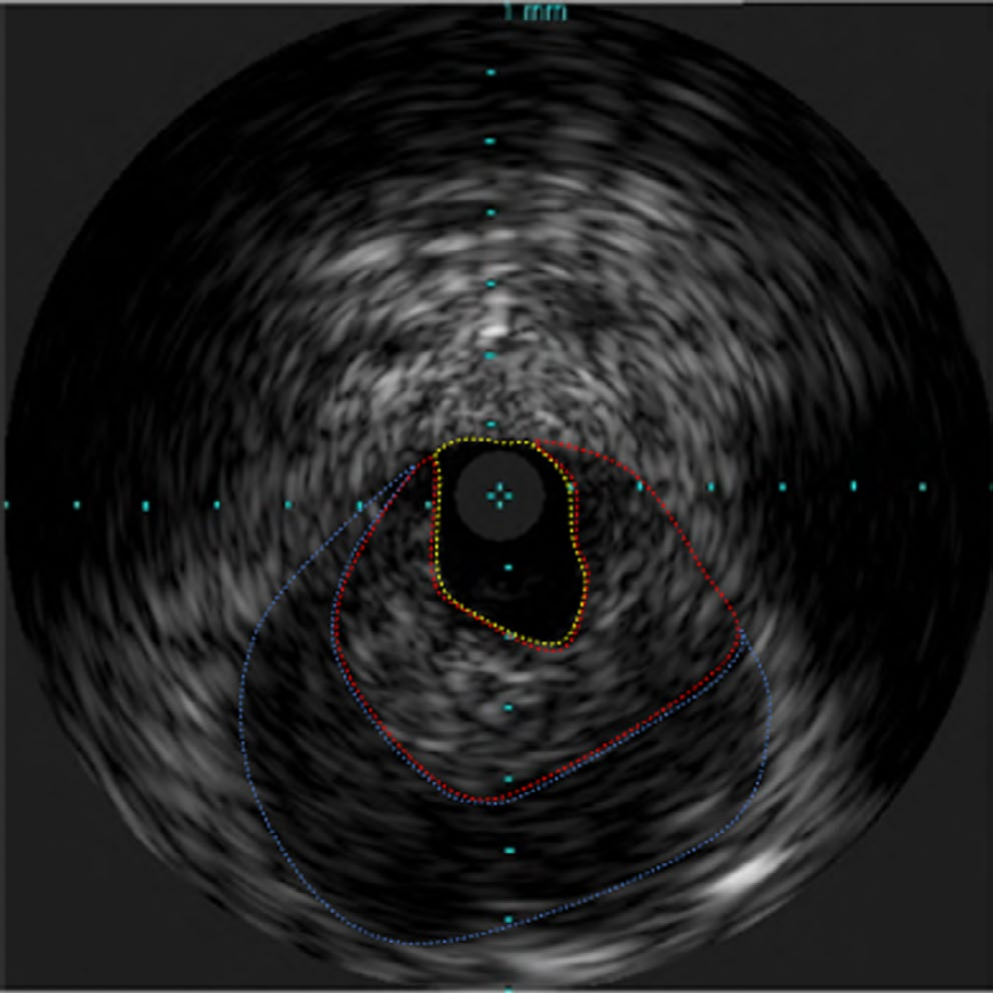
Intravascular ultrasound showed the true lumen was pressed upon by intramural hematoma. Yellow dotted line: true lumen. Red dotted line: hematoma. Blue dotted line: adventitia

**FIGURE 3:**
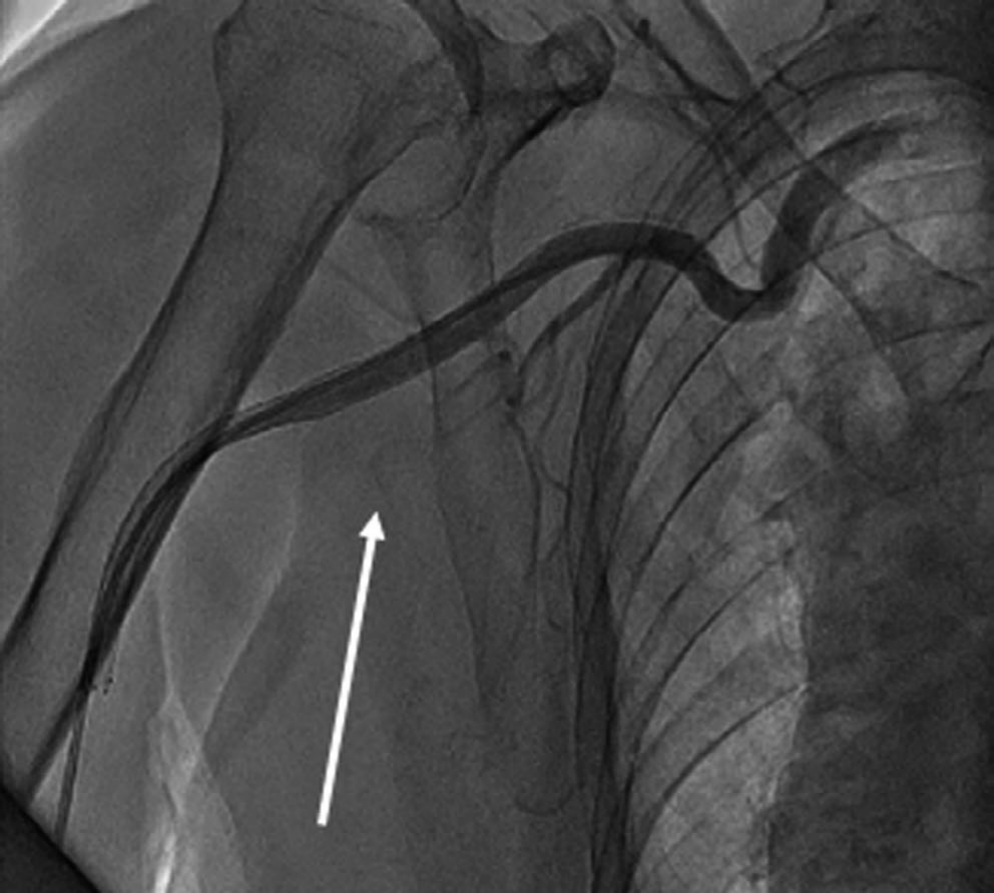
After the endovascular treatment, distal flow was achieved

A 7 Fr short sheath was placed in the right brachial artery. Angiography showed a defect from the axillary artery to the brachial artery. We attempted to penetrate the occluded lesion using 0.014‐inch guidewire with the support of 1.8/2.6 Fr microcatheter. Initially, the approach seemed successful, but intravascular ultrasound (IVUS) showed that the wire was in the subintimal lumen. We placed a 7 Fr sheath in her right common femoral artery and passed a 0.014‐inch guidewire through the lesion using the antegrade approach. IVUS showed that the guidewire was in the true lumen, and the true lumen was pressed by the intramural hematoma (Figure 2.). We extended the guidewire by using 0.014‐inch extension wire and passed it through with the support of a 7 Fr extension catheter from her right brachial artery. After exchanging the guidewire with 0.014‐inch support wire, we placed a bare‐metal stent (6.0 × 150 mm) and performed postdilatation with a balloon catheter (5.0 × 100 mm) (Figure 3). After the procedure, remarkable distal flow was achieved, and the procedure was terminated without any complications. Numbness and impaired skilled movement in her right arm were resolved after the EVT.

## 4 DISCUSSION

In this case, we faced difficulty in catheter maneuvering for her severe tortuous axillary artery during the PCI. We pushed against resistance in it several times and got the two guide catheters kinked. The sharp edge of kinked catheters and repeated excessive torqueing of catheters and the guidewire could cause artery injury. So, we should have changed artery access as soon as we faced difficulty in catheter maneuvering.

It has been reported that hematoma is often absorbed and dissipated spontaneously. However, in this case, the hematoma did not decrease and the true lumen was pressed upon by the IVUS finding.

The use of Amplatz‐shaped catheters has been reported to be a risk factor for catheter‐induced coronary artery dissection,^4^ but the association between the type of catheter and artery injury in the upper limb is still unclear.

The JR catheter is preferred for operability; however, severe vessel tortuosity may be complicated by axillary artery occlusion.^5^ Arterial tortuosity is associated with older age, female sex, high blood pressure, and other cardiovascular risk factors.^6^ For such cases, an operator should never push against resistance or cause excessive catheter torqueing.^7^ Arterial access changes should also be considered as soon as you feel uncomfortable with the catheter maneuver to prevent iatrogenic catheter‐induced complications.

## CONFLICT OF INTEREST

The authors declare that there is no conflict of interest.

## AUTHOR CONTRIBUTIONS

AU and ST: involved in preparing and writing the manuscript. ST: involved in the angiology. All authors: approved the final version of the case report for submission to *Clinical Case Reports*.

## ETHICAL APPROVAL

Informed consent was obtained from the patient for the publication.
